# Guidelines for developing and integrating 360-degree video in healthcare education

**DOI:** 10.12688/mep.20881.2

**Published:** 2025-05-29

**Authors:** Nynke de Jong, Ricardo G. Orsini, Dalena van Heugten–van der Kloet

**Affiliations:** 1 Department of Health Services Research, School of Health Professions Education, Faculty of Health, Medicine and Life Sciences, Maastricht University, Maastricht, The Netherlands; 2 Department of Surgery, Maastricht University Medical Centre+, Maastricht, The Netherlands; 3 Department Clinical Psychological Science, Faculty of Psychology and Neuroscience, Maastricht University, Maastricht, The Netherlands

**Keywords:** 360-degree video; Immersive video; VR in education; Use of technology in education; Healthcare education

## Abstract

The rapid growth of immersive virtual reality (VR) has gained widespread global attention in the field of education. In higher education within healthcare, VR has already been widely explored and employed. One specific form of virtual reality, 360-degree video, is regarded as a more user-friendly, realistic, and cost-effective alternative to other VR modalities, providing an immersive experience that requires less complex technology while still offering a high level of engagement in educational contexts. A 360-degree video is relatively easy to produce and can be seamlessly integrated into educational settings, serving a versatile and accessible tool for enhancing interactive learning experiences across various healthcare disciplines. We developed and integrated ten 360-degree videos, designed for viewing through head-mounted displays, to enhance educational practices in healthcare at Maastricht University, the Netherlands. In this article, we share guidelines for developing and integrating 360-degree videos into undergraduate and graduate healthcare programs, drawing on insights from our own experiences.

## Introduction

Over the past ten years, the rapid expansion of immersive virtual reality (VR) has attracted widespread global attention, alongside a rising interest in utilizing VR applications to advance various instructional design strategies within higher education settings ( Pellas *et al.*, 2021). A 360-degree video is considered a more user-friendly, realistic, and cost-effective alternative to VR ( Evens *et al.*, 2023). Students reported significantly higher levels of enjoyment, engagement, and immersion when experiencing 360-degree video compared to standard 2D video ( Kavanagh *et al.*, 2025). A systematic literature review identified five added values of using 360-degree videos as a teaching–learning medium: to increase learning motivation and interest, to learn in authentic and realistic learning scenarios, to enable immersive and interactive learning experiences, to offer multi-perspective observation opportunities, and to support individual learning ( Rosendahl & Wagner, 2024). There are several reasons for utilizing 360-degree videos in healthcare education. First, logistical challenges, such as the inability to organize field trips or internships and concerns over patient privacy, make it difficult to provide students with direct experiences in clinical settings ( De Jong *et al.*, 2020). Additionally, certain sensitive interactions, such as Advance Care Planning (ACP) conversations with palliative care patients, are typically conducted without the presence of students ( De Jong *et al.*, 2024). Another significant advantage of 360-degree videos is their ability to present an entire care trajectory within a relatively short time frame, such as following a patient from the onset of abdominal pain through to surgery, such as a laparoscopic cholecystectomy, without disrupting clinical practice ( Van Vlierden, 2024).

Evens, Empsen and Hustinx (2023) identified several positive effects of using 360-degree videos, watched by means of head-mounted displays, in educational settings. These videos were found to enhance mastery of learning content and improve learners' attitudes, while also significantly increasing participants’ sense of immersion and presence within the environment, creating the impression of being in another location. Moreover, 360-degree videos have been shown to foster empathy by enabling participants to approach situations from another person’s perspective. Some studies reported improvements in the self-reported confidence of future surgeons after engaging with 360-degree video content ( Evens *et al.*, 2023).

At the Faculty of Health, Medicine, and Life Sciences, we developed fifteen 360-degree videos, designed to be viewed through head-mounted displays, to enhance educational practices. These efforts were guided by a triangular framework that integrates stakeholders from education, research, and practice. The majority of the video scripts were developed by teachers, with support from researchers and input from practitioners. Students and health educators also actively contributed to the development process (co-creation), which was typically an iterative journey. The request to record the videos originated from practical needs within the educational field, aimed at addressing or clarifying specific challenges. The videos were recorded by a professional media production team. Examples of these 360-degree videos include *Technology in Home Care* for undergraduate Health Sciences (n=300) and Medical students (n=30) and *Surgery: Laparoscopic Cholecystectomy* for (under)graduate Medical students (n=300). In two instances, students from the Bachelor’s program in Global Studies wrote the scripts for *Intercultural Awareness* videos as part of a project. These videos were used to prepare undergraduate students in Health Sciences (n=50), Biomedical Sciences (n=45), and European Public Health (n=30) for their minor, and graduate Medical students (n=150) for their internship. Table 1 below presents three 360-degree video projects referenced in this article, including their rationale and one corresponding learning objective for each.

**Table 1.** Examples of 360-degree video projects: rationale and a learning objective.

360-degree video projects	Reasons for developing *	Learning objective *
Technology in Home Care	To ensure the privacy of patients/residents and manage the large number of students involved.	Students explain how policy influences the use of technology in home care.
Surgery: Laparoscopic Cholecystectomy	Prepare students for working effectively in the operating room, collaborating with different disciplines, and addressing sustainability issues.	Students describe the roles of the disciplines collaborating within an operating room.
Intercultural Awareness	Develop intercultural awareness and foster open dialogue about cultural differences.	Students describe two communication-related cultural differences between their visiting country abroad and their home country.

*The reasons/learning goals provided are not exhaustive.

In this article, we present the lessons learned from the development and integration of 360-degree videos in healthcare education based on these experiences.

## Tip 1

### Ensure that the support and organization for 360-degree videos are structured and secured

Establishing a well-structured and reliable framework and vision for the support and organization of 360-degree videos is essential for their effective and sustainable implementation. This requires a clear vision, along with both technical and organizational assistance. Regular maintenance plays a key role in ensuring long-term functionality and reliability to tackle simple problems. For instance, VR headsets require periodic updates, and the batteries in the controllers must be frequently replaced. In addition to maintaining the technical infrastructure, it is vital to establish processes for troubleshooting, reparations and user guidance. Hygiene standards must also be strictly upheld, including the regular cleaning of headsets, earphones, controllers, and lenses. Another important consideration is the location of the lessons. Should the VR headsets be transported across the building, or will the class be brought to the space where the headsets are already set up? By addressing all these aspects, educators and students are ensured a seamless experience, thereby maximizing the educational potential of this technology. Moreover, we think that it is essential to adopt a forward-looking perspective, which includes adapting the existing infrastructure (e.g. security, data storage), investing in new headsets, and ensuring sufficient budgetary resources to sustain the organization’s operations effectively. Think about a dedicated technical centre with a clear vision, this is essential to support the development, implementation, and maintenance of advanced technologies. Lastly, work together with a dedicated team of videographers, and editors to streamline the production process.

***Analysis phase***

The following three guidelines relate to the rationale for a 360-degree video, the sustainability and instructional design of the training (awareness and setup), module or curriculum in which the 360-degree video will be integrated. These considerations should be addressed *before* developing the 360-degree video.

## Tip 2

### Create a rationale for a 360-degree video

The main question to address when considering the integration of a 360-degree video in education is, ‘What is the specific necessity of a 360-degree video within this context, and why would a standard 2D video not suffice?’. Additionally, one should question if the benefit of usage outweighs the associated costs? The development of a 360-degree video can be justified by several key reasons, with immersion being a primary factor ( Rosendahl & Wagner, 2024). Both physical and mental engagement play a significant role in enhancing the viewer's experience. Another important aspect is encouraging students to actively explore their surroundings within the video. Unlike traditional 2D videos, which predetermine the viewer's focus by zooming in or directing attention to specific sections, a 360-degree video empowers the viewer to make their own decisions about where to look and engage. An example is the video on *Laparoscopic Cholecystectomy*. Students have the feeling they are present, have the best view and can focus on various aspect in the video such as details of the medical procedure, or communication or sustainability issues during the surgery. The immersive experience enhances student engagement and curiosity while facilitating a deeper understanding of complex concepts through diverse perspectives ( Jiang *et al.*, 2024).

## Tip 3

### Ensure the sustainable use of the 360-degree video

The initial consideration is whether a 360-degree video on the desired topic already exists. Prior to developing a 360-degree video, it is crucial to consider its potential integration into education. Key questions to address include: Will the 360-degree video remain relevant and usable across multiple academic years? Can it be effectively utilized in other modules or programs (within different faculties and healthcare institutions)? Conducting a pilot is not always necessary; instead, reviewing existing implementations by colleagues can provide valuable insights before deciding to proceed with production. Our video *Technology in Home Care* was developed collaboratively by teachers from various programs and (healthcare) institutions, enabling its applicability across multiple disciplines. The video evolved into a product of valorization.

Assuming the (higher) education institute has access to (compatible and up to date) VR headsets, it is essential to consider the support provided for teachers. If the video is used by a teacher only once a year, we recommend that they should not be burdened with the technology of the headset. In this case, a technical assistant should be available to assist the teacher during the educational session. However, if teachers are expected to use the video regularly, providing training for them becomes crucial, enabling them to independently facilitate the lesson. It is essential to ensure that teachers remain motivated and do not become discouraged to use the technology.

Another critical consideration is the sustainability of investments in hardware and equipment, particularly in light of the rapidly evolving technological landscape and the associated risk of obsolescence ( Ranieri *et al.*, 2022). It is advisable to select headsets that can be utilized effectively over multiple years.

## Tip 4

### The 360-degree video should be integrated into an active educational format

It is important to clarify that we did not create *interactive* 360-degree videos, such as integration of questions or collaborative learning in a 360-degree VR environment, in which students synchronously view and annotate a 360-degree video through a networked connection ( Davidsen *et al.*, 2024). The primary reason for this decision lies in the substantial costs associated with their production and the unstable internet connection during the use of VR headsets proved to be particularly challenging. Additionally, we chose not to develop 360-degree videos that would allow students to practice independently in their own time. Our *passive* 360-degree videos have been fully integrated into educational activities in which students *actively* participate. We strive to ensure the four learning key principles of Problem-Based Learning at Maastricht University (learning should be constructive, collaborative, contextual and a self-directed process) are upheld as much as possible when designing education ( Dolmans *et al.*, 2005). In the abdominal module, undergraduate medical students (into groups of four), meet the patient in the outpatient clinic (360-degree video) and, through the emergency department (360-degree video), ultimately follow the patient to the operating room (360-degree video). Each group assumes the role of the consulting physician in each phase of the patient journey, examining and discussing each step of the clinical trajectory supervised by their tutor. Most students expressed a preference for these educational formats over traditional methods, highlighting the value they found in the interactive components ( De Jong *et al.*, n.d.). Do not hesitate to use 360-degree videos in large groups (100+); instead, plan strategically and seek assistance when necessary. Moreover, like any other educational intervention, it necessitates a systematic approach encompassing its design, implementation, and evaluation to ensure its effectiveness (quality cycle).

***Development and recording phase***

Below are five guidelines related to development of the video and the recording process of a 360-degree video, offering practical insights to enhance the quality and effectiveness of the production.

## Tip 5

### Facilitate student acclimation to 360-degree videos

The development of the video begins with creating a detailed script and a corresponding storyboard. These serve as foundational tools to structure the narrative and visual elements. It is essential that the scenarios are authentic. Additionally, careful consideration is given to aspects such as the setting, colour scheme, and overall visual composition. These choices are guided by the educational objectives and the desired impact on the viewer, ensuring that the video effectively conveys its intended message. Moreover, it is important to provide students with an opportunity to acclimate to the video environment. Including a slightly longer introduction can help students orient themselves effectively before engaging with the main content. Similarly, a well-structured outro allows for a gradual transition out of the immersive experience, enhancing both comfort and comprehension. In our *Intercultural Awareness* video, the main character arrives by bicycle, approaches a building, and, at the video's conclusion, departs by bicycle.

If you believe that the target group requires additional support, it is advisable to provide students with a test video to watch beforehand. This allows them to adjust their glasses, familiarize themselves with the controllers, and acclimate to the visuals at their own pace.

## Tip 6

### Keep the 360-degree video under 15 minutes

The majority of 360-degree videos in education typically have a runtime ranging from five to fifteen minutes ( Evens *et al.*, 2023). In recent literature, researchers broadly agree that videos used in education are most effective for sustaining learner focus when presented in short segments ( Shephard, 2003). We created 360-degree videos with durations ranging from two to nineteen minutes. While no complaints were received from students regarding the video lengths, we recommend limiting video duration to 15 minutes or less. This recommendation is based on observations of some students reporting dizziness or nausea after viewing the 19-minute video. Additionally, comfort and proper adjustment of the VR headset appear to play a role in mitigating these issues. Factors such as the headset's fit, the pressure it exerts on the nose, and the quality of the headband contribute to the user’s overall experience and comfort during extended viewing sessions.

## Tip 7

### Think carefully about the position of the camera

The position of the camera plays a pivotal role, as it effectively assumes the function of the viewer's eye. According to Kavanagh *et al.* (2016), placing the camera at eye level enhanced the naturalness of the viewing experience. It fundamentally dictates what is visible and shapes how the viewer perceives and interacts with the digital environment.

For the 360-degree video on *Laparoscopic Cholecystectomy*, the camera was positioned on the ceiling right above the patient to provide a comprehensive overview of the entire operating room. In contrast, for conversations involving five or more participants, the camera was placed to the side rather than in the center (eye level). This choice was made to help the viewer identify the speaker more easily, as being centrally positioned can lead to confusion about who is speaking, difficulties in recognizing voices, and excessive head movement. By positioning the camera just above a person's head, we aimed to simulate a first-person perspective. Furthermore, the camera remained stationary throughout the recording to minimize the risk of inducing nausea.

The recorder must remain mindful that every detail within the environment will be captured. A thorough and deliberate walkthrough of the room or space is therefore essential. All unnecessary items will need to be cleared, and personal photographs or irrelevant objects will need to be replaced with those aligned with the scene's narrative.

## Tip 8

### Do not cut scenes and do not add subtitles (if not necessary)

Scenes in a 360-degree video should be recorded in a single take. This is essential because attempting to alter the footage post-capture can lead to distortions, such as areas becoming stretched or compressed, which ultimately undermine both the video quality and the viewer's overall experience. Our 360-degree videos are produced by stitching together footage from multiple cameras. If a portion of the video is cropped, it can accentuate the seams between the images, resulting in an unnatural and distracting appearance.

If you wish to translate your 360-degree video, as we did with our *Technology in Home Care* video, it is advisable to use dubbing, as subtitles may detract from the immersive experience by requiring viewers to read while simultaneously watching the video. Subtitling is, of course, feasible and may be necessary for various purposes. Zulkiewicz *et al.* (2020) chose to position the text at 0 and 180 degrees to ensure that a portion of the text remains visible from any viewpoint. It is essential to consider your target group with regard to accessibility.

## Tip 9

### Use ambisonics (full-sphere surround sound format)

Sound plays a crucial role in 360-degree videos, where ambisonics is particularly important. Ambisonics, an advanced form of surround sound, utilizes innovative technology that applies audio filters to replicate how the human ear perceives the distance and direction of sound. This creates a more realistic auditory experience; for example, when you turn your head away from a speaker, the volume of their voice decreases, just as it would in real life.

***Applying phase***

The final three guidelines concentrate on the implementation of 360-degree video within the educational context.

## Tip 10

### Ensure that the 360-degree video is accessible for offline viewing using a VR headset

Nothing is more frustrating than a 360-degree video malfunctioning in the VR headset, especially due to issues like unreliable internet network connectivity. Therefore, it is advisable to ensure that the video can be viewed offline within the headset, guaranteeing its consistent functionality. Another advantage is that large groups of students can be served simultaneously, without being restricted by location. This makes the content easily accessible for different groups on an ad hoc basis. Additionally, since students do not need to log in, potential privacy concerns are effectively avoided.

**Figure 1.**
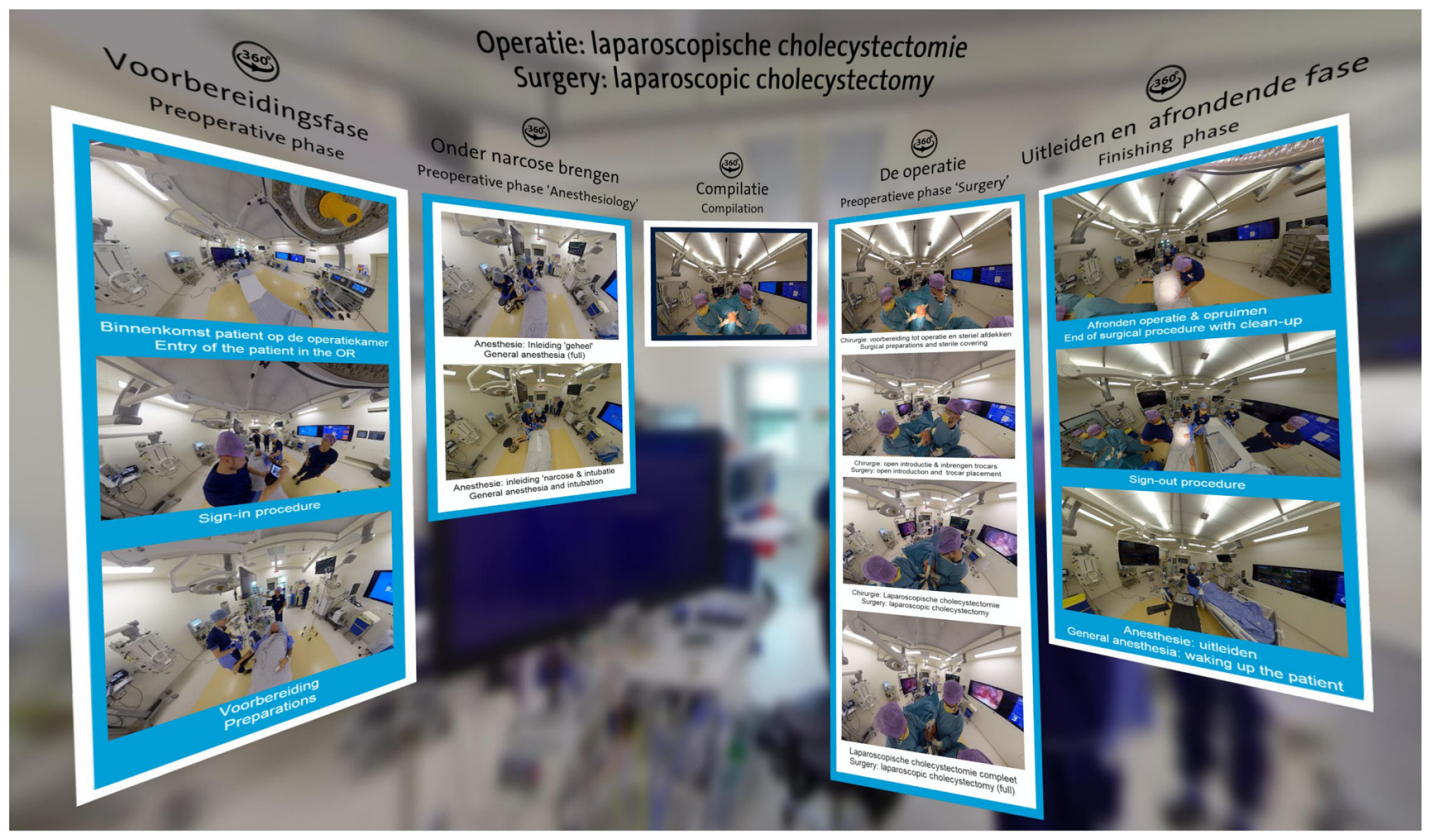
An example of the *Laparoscopic Cholecystectomy* project viewed in 3DVista using a VR headset.

For displaying our 360-degree videos offline, we use 3DVista on our Meta Quest 2 or 3 devices, an application that can be downloaded for free onto the headset. 3DVista is a software tool specifically designed for the creation of interactive virtual tours and immersive experiences. In the context of the *Laparoscopic Cholecystectomy* project, we developed a ‘bubble’ with various 360-degree videos (see Figure 1). Traditional 2D videos and other files, such as PDFs, can also be integrated into this bubble. This approach allows for the consolidation of all relevant information in one accessible space.

## Tip 11

### Bring the laptop on which the 360-degree video is stored

When teaching with VR headsets and students are set to watch a 360-degree video, it is advisable to also have the video available on a laptop. Some students may indicate in advance that they do not wish to wear a headset due to conditions such as vision impairments. These students can view the video on the laptop and explore the environment using the mouse. Additionally, students who experience motion sickness or anticipate it beforehand can also watch the video on the laptop. Based on our experience, approximately 1%–3% of the students reports feeling nauseous or dizziness ( De Jong *et al.*, 2025), a common side effect of immersive technologies ( Schott & Marshall, 2021). 

It may be beneficial to have a laptop available when using certain VR headsets (e.g., not the Meta Quest headset), particularly if a student accidentally deletes a video from the headset. In such cases, the laptop allows for the quick restoration of the video onto the device.

## Tip 12

### Provide an assignment for watching the 360-degree video

Providing a clear and detailed assignment on how to engage with 360-degree video content is crucial for optimizing the immersive learning experience. Without explicit guidance, students may become disoriented or fail to engage with the video effectively, leading to a diminished educational experience. For example, it is important to clarify the specific tasks or objectives the students should focus on during the video. Are they expected to answer questions based on the content, or should they be identifying key elements, recognizing patterns, or analysing certain aspects of the environment? In our *Intercultural Awareness* video, students first attended a theoretical lecture on communication. Following this, the 360-degree video was designed to help students recognize key concepts, which they then discussed in small groups before engaging in a plenary discussion, thus embedding the 360-degree video within the educational setting. Clear instructions provide structure and direction, ensuring that students remain engaged and that the learning outcomes are effectively achieved.

## Conclusion

The development and integration of 360-degree videos in healthcare education offer transformative opportunities, but their successful implementation requires strategic planning and consideration across several dimensions.

A robust framework for technical and organizational support is essential to ensure long-term functionality and reliability. Sustainability is crucial, requiring investments in VR technology that anticipate rapid advancements and obsolescence, with durable equipment and adequate teacher training ensuring long-term viability. The method of recording a 360-degree video is key, with the camera position being particularly significant, while also considering the accessibility of the produced video. The design of the content must align with educational objectives. Immersive experiences enhance engagement and facilitate deeper understanding when integrated into active educational formats. Practical considerations during the session, such as ensuring videos are accessible offline, accommodating students who may experience discomfort, and providing explicit assignments, further optimize the learning experience and accessibility. Ultimately, embedding 360-degree videos within a sustainable and interactive framework not only maximizes their educational potential but also minimizes challenges. By adopting a systematic approach to design, implementation, and evaluation, teachers can leverage this technology to create meaningful, immersive learning environments that align with institutional goals and resources.

## Ethics and consent

Ethical approval and consent were not required.

## Data Availability

No data associated with this article.
