# Supplementary material for: Guidelines for developing and integrating 360-degree video in healthcare education
Source: MedEdPublish (2016). 2025 May 29;15:9. Originally published 2025 Mar 25. [Version 2] doi: 10.12688/mep.20881.2 (PMC12134730; doi:10.12688/mep.20881.2)
Supplement: Peer review history

# Reviewer report: Reviewer response for version 2

**DOI:** 10.21956/mep.22585.r41951

**Published:** 2025-06-03

**Authors:** Burkhard Wünsche

**Affiliations:** 1 The University of Auckland, Auckland, Auckland, New Zealand

The authors have incorporated many of the suggestions from the reviews of earlier versions and the current version provides an improved and more nuanced discussion of the topic with more citations providing evidence. I am happy to recommend indexing this revised version.

If evidence from practice is presented, are all the underlying source data available to ensure full reproducibility?

No

Is the topic of the practical tips discussed accurately in the context of the current literature

Partly

Are all factual statements correct and adequately supported by citations?

Partly

Are the conclusions drawn balanced and justified on the basis of the presented arguments?

Partly

Are arguments sufficiently supported by evidence from the published literature and/or the authors’ practice?

Partly

Reviewer Expertise:

AR/VR, education, computer graphics, HCI

I confirm that I have read this submission and believe that I have an appropriate level of expertise to confirm that it is of an acceptable scientific standard.

# Reviewer report: Reviewer response for version 1

**DOI:** 10.21956/mep.22360.r41374

**Published:** 2025-05-02

**Authors:** Burkhard Wünsche

**Affiliations:** 1 The University of Auckland, Auckland, Auckland, New Zealand

This article offers some interesting and useful guidelines for integrating 360 degree videos into healthcare education. The paper is in general well written and easy to understand.

**Below are a couple of suggestions how the article could be improved:**

1. Please reference previous paper providing guidelines on creating 360 degree videos for education, e.g., https://dl.acm.org/doi/10.1145/3010915.3011001 and https://games.jmir.org/2020/1/e15422 In particular, you might want to discuss your guidelines in context of the guidelines given in the above articles.
2. The paper would benefit from a review of previous work on using 360 degree videos for education, e.g., why is it used, how is it used, what are its benefits There are even some review papers about this topic, e.g., https://ieeexplore.ieee.org/document/9384236/ https://link.springer.com/article/10.1007/s11528-019-00474-3
https://www.sciencedirect.com/science/article/abs/pii/S1931720417302945
https://link.springer.com/article/10.1007/s10639-022-11549-9
https://www.tandfonline.com/doi/full/10.1080/15391523.2021.1928572
https://linkinghub.elsevier.com/retrieve/pii/S0360131523001781
  https://www.sciencedirect.com/science/article/abs/pii/S0260691723001806?via%3Dihub
3. Please discuss in more detail why 360 degree video was chosen. For example, it is true that 360 video is more engaging, but it can also be more distracting – especially, if the surrounding environment does not contain any information assisting learning. The following paper provides some results about advantages and disadvantages of 360 videos compared to normal videos: https://www.mdpi.com/2079-9292/14/9/1830
4. I was hoping to see some student feedback, e.g., what did they like and dislike about the 360-degree videos? Where did it help with learning and why/how? Where did the videos not help and why not? I believe this information would be of most value to a reader of this article.
5. I found the 12 tips given in this paper plausible and useful. Several of them correspond with guidelines proposed in previous research. However, it seems the videos used in this research covered a wide range of different application scenarios and I wonder whether the tips really apply equally to all these scenarios. For example, I would have thought that a video demonstrating a surgery if made in a different way (having a different focus) than a video showcasing the climbing of a mountain. It would be good to talk about a few of the videos, their different learning goals, learning barriers students struggle with, and how this information was reflected in the content and recording of the video.

If evidence from practice is presented, are all the underlying source data available to ensure full reproducibility?

No

Is the topic of the practical tips discussed accurately in the context of the current literature

Partly

Are all factual statements correct and adequately supported by citations?

Partly

Are the conclusions drawn balanced and justified on the basis of the presented arguments?

Partly

Are arguments sufficiently supported by evidence from the published literature and/or the authors’ practice?

Partly

Reviewer Expertise:

AR/VR, education, computer graphics, HCI

I confirm that I have read this submission and believe that I have an appropriate level of expertise to confirm that it is of an acceptable scientific standard, however I have significant reservations, as outlined above.

## References

[1] Creating 360° educational video: a case study. In Proceedings of the 28th Australian Conference on Computer-Human Interaction (OzCHI '16) Sam Kavanagh, Andrew Luxton-Reilly, Burkhard Wüensche, and Beryl Plimmer. *Association for Computing Machinery, New York, NY, USA* .2016. 34–39; 10.1145/3010915.3011001

[2] : Using 360-Degree Video as a Research Stimulus in Digital Health Studies: Lessons Learned. *JMIR Serious Games* .2020;8(1) : 10.2196/15422 e15422 10.2196/15422 31904577 PMC6971508

[3] : The Potential of 360° Virtual Reality Videos and Real VR for Education-A Literature Review. *IEEE Comput Graph Appl* .2021;41(4) : 10.1109/MCG.2021.3067999 76-89 10.1109/MCG.2021.3067999 33755559

[4] : Educational 360-Degree Videos in Virtual Reality: a Scoping Review of the Emerging Research. *TechTrends* .2020;64(3) : 10.1007/s11528-019-00474-3 404-412 10.1007/s11528-019-00474-3

[5] : 360° Operative Videos: A Randomised Cross-Over Study Evaluating Attentiveness and Information Retention. *J Surg Educ* .2018;75(4) : 10.1016/j.jsurg.2017.10.010 993-1000 10.1016/j.jsurg.2017.10.010 29122571

[6] : 360° videos in education – A systematic literature review on application areas and future potentials. *Education and Information Technologies* .2024;29(2) : 10.1007/s10639-022-11549-9 1319-1355 10.1007/s10639-022-11549-9

[7] : A review of research on 360-degree video and its applications to education. *Journal of Research on Technology in Education* .2022;54(5) : 10.1080/15391523.2021.1928572 784-799 10.1080/15391523.2021.1928572

[8] : A meta-analysis on learning from 360° video. *Computers & Education* .2023;206: 10.1016/j.compedu.2023.104901 10.1016/j.compedu.2023.104901

[9] : Come and see through my eyes: A systematic review of 360-degree video technology in nursing education. *Nurse Educ Today* .2023;128: 10.1016/j.nedt.2023.105886 105886 10.1016/j.nedt.2023.105886 37390520

[10] : Evaluating the Use of 360-Degree Video in Education. *Electronics* .2025;14(9) : 10.3390/electronics14091830 10.3390/electronics14091830

# Reviewer report: Reviewer response for version 1

**DOI:** 10.21956/mep.22360.r41372

**Published:** 2025-04-23

**Authors:** Jacob Gorm Davidsen

**Affiliations:** 1 Aalborg University, Aalborg, Denmark

de Jong *et al.* (2025) offer a timely and practical contribution by outlining design guidelines for the integration of 360-degree video in healthcare education. Their work provides useful direction for institutions and educators seeking to adopt immersive video formats, particularly for observational learning and self-paced, asynchronous engagement. The emphasis on realism, ease of use, and alignment with PBL structures is commendable and grounded in current institutional needs. However, the paper frames 360VR primarily as a passive media experience—focusing on viewing, observation, and reflection—rather than considering how such video-based environments can become interactive and collaborative spaces for learning. This perspective does not fully account for recent developments in networked, multi-user immersive platforms that enable participants to engage in real-time discussion, annotation, movement, and co-analysis within shared 360VR spaces.

 In contrast, Davidsen *et al.*, (2022, 2024) demonstrates how 360VR can be used to support collaborative clinical training, in which groups of learners not only observe, but jointly interpret, annotate, and interact within the immersive environment. The platform CAVA360VR² was developed precisely to support such collaborative, embodied, and synchronous learning activities—grounded in sociocultural learning theory and designed to operationalize concepts like mutual and embodied awareness, shared marks and references, and embodied forms of participation.

 While de Jong *et al.* rightly focus on pragmatic integration and usability, future frameworks for 360VR in healthcare education should consider the growing potential of interactive, synchronous collaboration. Such an expansion of scope would not only align with current advances in immersive technology, but also deepen the educational value of 360VR beyond its role as a tool for passive observation.

If evidence from practice is presented, are all the underlying source data available to ensure full reproducibility?

Partly

Is the topic of the practical tips discussed accurately in the context of the current literature

Yes

Are all factual statements correct and adequately supported by citations?

Partly

Are the conclusions drawn balanced and justified on the basis of the presented arguments?

Yes

Are arguments sufficiently supported by evidence from the published literature and/or the authors’ practice?

Yes

Reviewer Expertise:

Collaboration in digital context - especially collaborative 360VR.

I confirm that I have read this submission and believe that I have an appropriate level of expertise to confirm that it is of an acceptable scientific standard.

## References

[1] : Collaborative 360° virtual reality training of medical students in clinical examinations. *Med Educ Online* .2024;29(1) : 10.1080/10872981.2024.2412398 2412398 10.1080/10872981.2024.2412398 39363154 PMC11451289

[2] : 360VR PBL: A New Format of Digital Cases in Clinical Medicine. *Journal of Problem Based Learning in Higher Education* .2022; 10.54337/ojs.jpblhe.v10i1.7097 10.54337/ojs.jpblhe.v10i1.7097
